# A guide for pre-procedural imaging for transcatheter aortic valve replacement patients

**DOI:** 10.1186/s13741-020-00165-1

**Published:** 2020-11-26

**Authors:** Tjörvi E. Perry, Stephen A. George, Belinda Lee, Joyce Wahr, Darrell Randle, Garðar Sigurðsson

**Affiliations:** 1 Department of Anesthesia, Division of Cardiothoracic Anesthesia, University of Minnesota, 420 Delaware St SE, MMC 294, Minneapolis, MN 55455 USA; 2 Department of Cardiology, Regions Hospital Heart Center, 640 Jackson Street, Saint Paul, MN 55101 USA; 3 Department of Anesthesia, Preoperative Assessment Center, University of Minnesota, 420 Delaware St SE, MMC 294, Minneapolis, MN 55455 USA; 4 Department of Cardiology, University of Minnesota, 420 Delaware St. SE, MMC 207, Minneapolis, MN 55455 USA

**Keywords:** TAVR, Imaging, Preoperative assessment, Echocardiography

## Abstract

Safe and accurate pre-procedural assessment of cardiovascular anatomy, physiology, and pathophysiology prior to TAVR procedures can mean the difference between success and catastrophic failure. It is imperative that clinical care team members share a basic understanding of the preprocedural imaging technologies available for optimizing the care of TAVR patients. Herein, we review current imaging technology for assessing the anatomy, physiology, and pathophysiology of the aortic valvular complex, ventricular function, and peripheral vasculature, including echocardiography, cardiac catheterization, cardiac computed tomography, and cardiac magnetic resonance prior to a TAVR procedure. The authorship includes cardiac-trained anesthesiologists, anesthesiologists with expertise in pre-procedural cardiac assessment and optimization, and interventional cardiologists with expertise in cardiovascular imaging prior to TAVRs. Improving the understanding of all team members will undoubtedly translate into safer, more coordinated patient care.

## Introduction

The prevalence of aortic stenosis (AS) is rapidly increasing, with valve calcification being the most common cause in developed countries (Roberts and Ko 2005). An estimated 3% of individuals over the age of 75 years have AS (Manning 2013). This translates into as many as 630,000 people with AS in the USA alone. Though the presentation of AS is insidious, once symptoms occur, the disease progression is rapid. With conservative therapy, severe, symptomatic AS is associated with a mortality of 50% at 1 year (Leon et al. 2010). Complicating the care of these patients is the high incidence of frailty which increases the risk of surgical aortic valve replacement (SAVR) (Arnold et al. 2016). Although there are a number of definitions, frailty has been found to be present in almost 60% of patients with severe AS at the time of presentation (Rowe et al. 2014). As a result, an estimated 31.8% of patients with AS are not candidates for SAVR (Iung et al. 2003).

Minimally invasive transcatheter aortic valve replacement (TAVR) is a seemingly ideal solution for this high-risk surgical population with correspondingly high-risk disease. The first human transcatheter aortic valve was placed in 2002. In the 20 years since, over 300,000 TAVRs have been performed in 65 countries (Cribier 2016). In 2010, the first PARTNER B study demonstrated that balloon-expandable TAVR decreased mortality from 50.7 to 30.7% at 12 months when compared with conventional non-surgical treatment (Leon et al. 2010). In 2011, balloon-expandable TAVR was shown to be non-inferior to SAVR in high-risk patients (Smith et al. 2011). Subsequent studies in 2016 (Leon et al. 2016) and 2017 (Reardon et al. 2017) demonstrated non-inferiority of both balloon-expandable valves and self-expanding valves with respect to mortality and disabling stroke in intermediate-risk patients when compared to SAVR at 2 years. A recent outcome study demonstrated non-inferiority of TAVR to SAVR, and no structural valve deterioration at 5 years (Mack et al. 2015), and even more recently, the PARTER 3 investigators demonstrated a significantly lower rate of the composite outcomes of death, stroke, and rehospitalization at 1 year in low-risk TAVR patients when compared with SAVR patients (Mack et al. 2019). Notably, it is anticipated that improvements in valve and valve deployment technology coupled with improved operator experience may increase the advantage of TAVR over SAVR even further (Leon et al. 2016).

In 2014, the American College of Cardiology published the American Heart Association/American College of Cardiology (AHA/ACC) Guidelines for the Management of Patients with Valvular Heart Disease (Nishimura et al. 2014) with a Focused Update in 2017 (Nishimura et al. 2017). This was supplemented with the ACC Expert Consensus Decision Pathway for Transcatheter Aortic Valve Replacement in 2017 (Otto et al. 2017). These clinical policy documents focus on “concise decision pathways and/or key points of care.” (Otto et al. 2017) The policies emphasize a systematic approach to the valve replacement patient, accurate diagnosis and staging of the AS, and consideration of the underlying risk for SAVR including the Society of Thoracic Surgeon (STS) Predicted Risk of Mortality score (O'Brien et al. 2009), frailty, organ system dysfunction, and procedural impediments (Otto et al. 2017). Most importantly, these policies all emphatically stipulate the need for the Heart Valve Teams (class I recommendation) that include cardiology valve experts, cardiovascular imaging experts, interventional cardiologists, cardiothoracic surgeons, cardiothoracic anesthesiologists, and valve clinic care coordinators (Nishimura et al. 2014).

As the indications broaden to include intermediate and low-risk patients, and the device technology continues to improve, the TAVR procedure is becoming increasingly more common in both the academic and private practice medical setting. Along with major advancements in device and deployment technology, peri-procedural imaging is also advancing. With higher resolution imaging, three-dimensional reconstruction, and image integration, our ability to accurately evaluate anatomic structures from the aortic valvular complex (AVC) to the ilio-femoral vasculature is making the TAVR procedure safer and more effective. As increasingly more institutions adopt the multidisciplinary Heart Valve Team model, cross-disciplinary education becomes imperative. In this review, we aim to outline the current standard of practice for pre-procedural imaging for the TAVR procedure. The authorship of this review includes cardiac-trained anesthesiologists, anesthesiologists with expertise in pre-procedural cardiac assessment and optimization, and cardiologists with expertise in cardiovascular imaging prior to and during TAVRs. Improving the understanding of all team members will undoubtedly translate into safer, more coordinated patient care.

## Echocardiography

Accurate and adequate imaging prior to a TAVR procedure ensures safe and effective device deployment, giving the patient the highest likelihood for long-term benefit. The majority of patients will undergo transesophageal echocardiography (TTE) as an initial evaluation for symptoms of AS that might include shortness of breath, fatigue, and chest discomfort. Echocardiographic assessment of the aortic root and surrounding structures gives the care team a general sense for the anatomy and function of the aortic valvular complex, extent and location of surrounding calcification, severity of aortic valve stenosis, and assessment of ventricular function (Baumgartner et al. 2009).

**Fig. 1:**
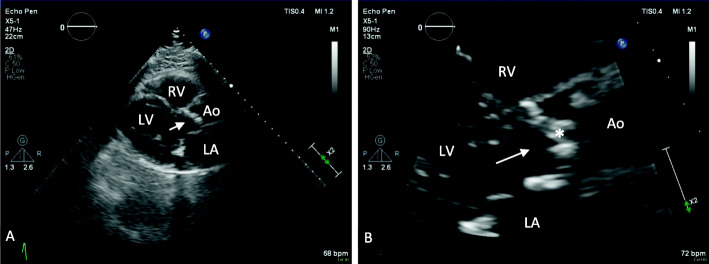
Trans-thoracic parasternal long-axis echocardiographic view. **a** This example demonstrates a hypertrophic left ventricle (LV) and calcified aortic valve (arrow in the left ventricular outflow tract pointing toward aortic valve. **b** This example demonstrates a severely calcified aortic valve (*)

**Fig. 2:**
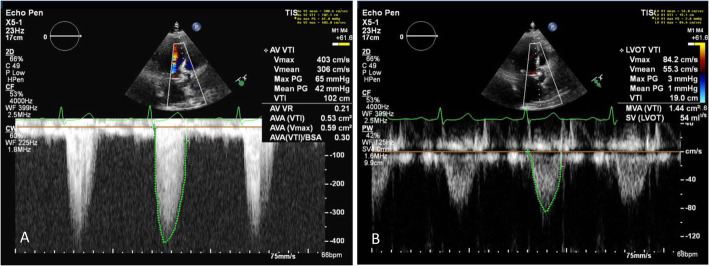
Blood flow velocities and pressure gradients are calculated by tracing the velocity time integral (green). **a** Aortic valve (AV). **b** Left ventricular outflow tract (LVOT)

A normal aortic valve area (AVA) is 3 to 4 cm^2^. Aortic valve stenosis becomes severe when the area is reduced to about 25% of normal (example provided in Fig. 1). The American Society of Echocardiography (ASE) defines severe AS as a peak transaortic valve blood flow velocity greater than 4 m/s, a mean transaortic valve pressure gradient greater than 40 mm Hg, or AVA less than 1 cm^2^ in the presence of normal left ventricular function (example provided in Fig. 2). Using echocardiography, the continuity equation is used to calculate the effective AVA by measuring the aortic and left ventricular outflow tract (LVOT) velocity-time integrals and LVOT cross-sectional area. The continuity equation is based on the principle that the left ventricular stroke volume ejected through the LVOT is equal to the stroke volume ejected through the aortic valve, and by knowing the cross-sectional area of the LVOT, can be used to calculate the cross-sectional area of the aortic valve. The AVA can be indexed to body surface area (BSA), but this becomes problematic and inaccurate with obese patients. A dimensionless index (DI) is the ratio of the velocity of blood flow in the LVOT to the velocity of blood flow across the aortic valve. When this ratio becomes less than 0.25, the aortic valve stenosis is considered severe (Otto et al. 1986).

Assessment of left ventricle size, thickness, and function is also important to evaluate an initial echocardiographic examination. Long-standing AS with a fixed afterload causes significant remodeling of the left ventricle (LV), leading to concentric hypertrophy, manifesting as elevated left ventricular filling pressures, and diastolic dysfunction. Over time, patients may develop systolic dysfunction. While reduced left ventricular ejection fraction (LVEF) is associated with poor outcomes after SAVR, the prognostic data for TAVR is still equivocal (Moat et al. 2011; Tamburino et al. 2011; Di Mario et al. 2013).

It is not uncommon for patients who present with AS to also present with additional or associated valvular lesions. An estimated 80% of patients with severe AS will have some aortic regurgitation (AR) (Baumgartner et al. 2009). In the vast majority of patients with associated AR, the severity is mild or moderate and does not significantly impact echocardiographic measurements. This is not the case, however in patients with severe AR in whom the mean gradient across the stenotic aortic valve and the maximum velocity across the same area will be overestimated due primarily to increased blood flow across the aortic valve. Functional mitral regurgitation (MR) is common in patients with AS due primarily to LV remodeling and chronically elevated left ventricular filling pressures. Ischemic MR is also possible due to the high concomitance of coronary artery disease (CAD) in the AS population. About 20% of patients with severe AS have moderate to severe MR, which improves after TAVR in about 50% of patients (Bedogni et al. 2013).

A detailed discussion of low flow, low gradient (LF-LG) AS is beyond the scope of this review. For a comprehensive review, the authors highly recommend the review by Pibarot and colleagues (Pibarot and Dumesnil 2012a). Briefly, however, grading the severity of AS in patients who present with LF-LG across the aortic valve can be challenging. Patients can present with either normal or reduced LVEF (< 40%). In both cases, a decreased transvalvular gradient relative to the severity of the AS is due to reduced flow across the aortic valve (Pibarot and Dumesnil 2012a). In patients with LF-LG AS and depressed left ventricular function, truly severe AS versus pseudo-severe AS can be distinguished by a dobutamine stress echo (Class IIa recommendation). Patients with truly severe AS will have a greater than 20% increase in stroke volume, i.e., flow, in response to a dobutamine infusion, and a corresponding increase in peak transvalvular velocities greater than 4 m/s and mean transvalvular gradient greater than 40 mmHg. Patients with LF-LG AS have been shown to have functional improvement at 1 year after TAVR although not significant improvement in LV function (Lauten et al. 2012). Those with pseudo-severe AS will have less than a 20% increase in flow across their aortic valve and are best managed medically. Patients who have severe AS, normal left ventricular function, but profound concentric left ventricular hypertrophy and an obliterated left ventricular cavity may not be able to generate enough left ventricular stroke volume (stroke volume index less than 35 ml/m^2^) to fully open the aortic valve (Hachicha et al. 2007). A lower than expected transvalvular gradient in the setting of normal left ventricular function has been termed paradoxical LF-LG. Paradoxical LF-LG severe AS is not common, and optimal management remains controversial (Vahanian et al. 2012; O'Sullivan et al. 2013).

There may be instances when TTE is not sufficient for assessing aortic valve anatomy and function in preparation for a TAVR. This may be the case in obese individuals or patients who otherwise have poor echocardiographic transthoracic windows. This often results in echocardiographic results that are not consistent with the clinical picture for a given patient and require further investigation of the aortic valve. In some instances, the team may opt for transesophageal echocardiography (TEE), a valuable, albeit more invasive, imaging option. TEE is most helpful when acoustic windows limit TTE. TEE allows a high definition view of the aortic valve and can offer planimetry to obtain valve area. Doppler can be challenging with TEE, but transgastric views can be particularly beneficial in order to line up Doppler with the blood flow across the aortic valve. Others may opt for cardiac computed tomography or magnetic resonance, which are discussed in more detail in subsequent sections. It is important to keep in mind that if TTE cannot be used during pre-operative evaluation, this imaging option may be limited during the procedure itself. This limitation may, in turn, dictate anesthetic management as intra-procedural utilization of TEE will likely necessitate a general anesthetic. During the procedure, TEE can provide a very accurate assessment of paravalvular leak.

## Cardiac catheterization

**Fig. 3:**
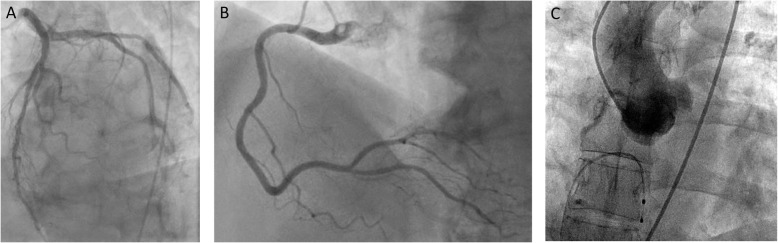
Angiogram demonstrating an example of non-obstructive coronary artery disease in **a** the left main, circumflex, and left anterior descending coronary arteries; **b** the right coronary artery; and **c** aortogram demonstrating three sinuses of Valsalva

Preprocedural coronary angiogram and right heart cardiac catheterization (example provided in Fig. 3, with hypothetical results in Table 1) is the standard of care in all patients presenting with AS being considered for surgical or transcatheter AV replacement. This ensures and understanding of pulmonary pressures prior to the procedure to know if further precautions are necessary. Vascular access can be evaluated if necessary during invasive angiography. Given that 25–50% of patients with AS have clinically significant CAD (Exadactylos et al. 1984), all patients being considered for TAVR should undergo a coronary angiogram. Depending on the severity and extent of coronary artery disease, the patient may either undergo percutaneous coronary intervention (PCI) prior to their TAVR procedure or surgical AVR with concomitant coronary artery bypass grafting. Regarding TAVR, there is a substantial debate in the literature as to whether CAD should have PCI prior to TAVR. In general, most operators will consider left main or proximal LAD PCI, but often other lesions can be deferred. An additional consideration to consider prior to valve replacement is whether coronary access will be altered after TAVR. Coronary ostial occlusion after TAVR deployment is a known risk and can present as an early (Sanchez and SJY 2016) or late complication (Ramirez et al. 2017). The risk for coronary occlusion is dependent on the entire aortic root anatomy as discussed later in this review. Even in the absence of coronary occlusion, the presence of a TAVR with its wire cage can increase the difficulty of coronary access. For this reason, many interventionalists opt to treat significant CAD lesions prior to performing TAVR.

**Table 1:** Hemodynamic results from left and right heart catheterization

LHC	
Aortic root pressures (mean pressure), mmHg	160/81(91)
LVP	Not measured
FA saturation, %	98
RHC	
mRAP	7 mmHg
RVP	27/7 mmHg
PAp (meanPAp)	27/17 (30) mmHg
PCWP	10 mmHg
Fick CO/CI	5.5/2.8
PA saturation	59%

*LHC* left heart catheterization, *RHC* right heart catheterization, *LVP* left ventricular pressure, *FA* femoral artery, *mRAP* mean right atrial pressure, *RVP* right ventricular pressure, *PAp* pulmonary artery pressure, *PCWP* pulmonary capillary wedge pressure, *CO* cardiac output, *CI* cardiac index, *PA* pulmonary artery

Left heart catheterization can also be considered prior to valve replacement. Direct measurement of LV/aortic pressures can be particularly helpful if there is a discrepancy of clinical presentation with echocardiography obtained hemodynamics. Using the Gorlin equation, an aortic valve area is calculated from the gradient measured directly between the left ventricle and aorta, as follows: AVA (cm^2^) = CO (L/min)/√(mean pressure gradient across AV (mm Hg)). An AVA less than 1 cm^2^ is considered severely stenotic. Once indexed for body surface area, an AVA less than 0.6 cm^2^ is considered severe. Crossing the aortic valve during the preprocedural catheterization not only allows for an accurate calculation of the severity of aortic valve stenosis, but also ensures the feasibility of crossing the valve during the TAVR procedure itself. This should be done judiciously, as any attempt to cross the aortic valve in retrograde fashion however, places the patient at risk for embolic stroke. A prospective randomized study of 152 patients suggested that as many as 22% in whom retrograde catheterization of the aortic valve was attempted had focal diffusion-imaging abnormalities in a pattern consistent with acute cerebral embolic events after the procedure. Three percent of these patients had clinically apparent neurological deficits (Omran et al. 2003). To reduce the risk of embolic stroke, some have suggested that the time of manipulation should be limited (usually less than 10 min) and only attempted if feasible. The inability to cross the aortic valve at the time of initial workup is not, however, an absolute contraindication to TAVR.

Catheterization of the right heart gives valuable information about pulmonary artery pressure, pulmonary vascular resistance, and cardiac output. Pulmonary hypertension, defined as mean pulmonary artery pressure (PAp) greater than or equal to 20 mmHg in the presence of normal pulmonary capillary wedge pressure, is associated with an increased risk of long-term mortality in patients undergoing TAVR (Alushi et al. 2018). Pulmonary hypertension from higher left-sided heart pressures seen with AS should theoretically be reduced once the new aortic valve is in place. Any other causes of elevated pulmonary artery pressures or pulmonary vascular resistance require further investigation. Thermodilution cardiac output measurements can be substituted in the Gorlin equation to calculate the AVA, while measured stroke volume provides a stroke volume index (SVI). In the PARTNER trial, patients with low SVI (< 35 ml/m^2^) had an increased risk of mortality (Herrmann et al. 2013).

During the early days of TAVR, when the device and delivery profiles were larger, a detailed understanding of the peripheral vasculature was imperative. Correctly sizing the ilio-femoral arteries to the deployment catheters could mean the difference between a successful procedure and catastrophic peri-procedural hemorrhage. The predominant modality for peripheral measurement is CT, but ilio-femoral angiography also allows for grading of the calcification of the arteries (Eltchaninoff et al. 2009) and measurement of the lumen size. If the femoral vessels are less than 6 mm in diameter, or contain significant atherosclerosis, calcification, or tortuosity, an alternative vascular approach might be considered. As TAVR device technology has improved and catheter profiles have decreased in size, the ilio-femoral arteries have become a viable access sites in the majority of TAVR procedures. Noninvasive assessment using CT has, in large part, replaced peripheral angiography as the definitive tool for assessing vascular anatomy and severity of calcification and for determining the access site for the procedure itself (Zaman et al. 2015). If the patient is not a candidate for the femoral approach, an alternative approach may be considered including the subclavian, axillary or carotid arteries, or the inferior vena cava. A transaortic or transapical approach is generally reserved for circumstances where these alternative approaches are not feasible or are unsafe.

## Multi-detector computed tomography (MDCT)

**Fig. 4:**
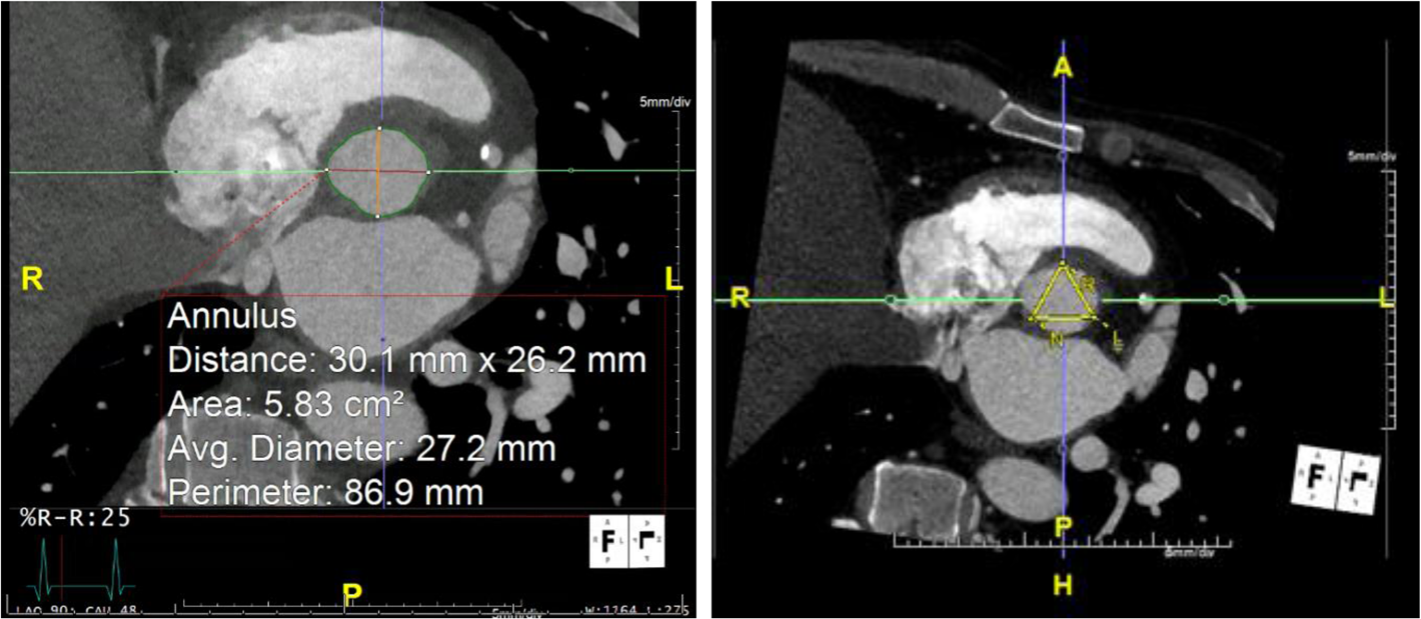
Multi-detector computer tomography (MDCT) example of aortic annular dimensions including the average aortic valve diameter (27.2 mm), area (5.83 cm^*2*^), and perimeter (86.9 mm)

**Fig. 5:**
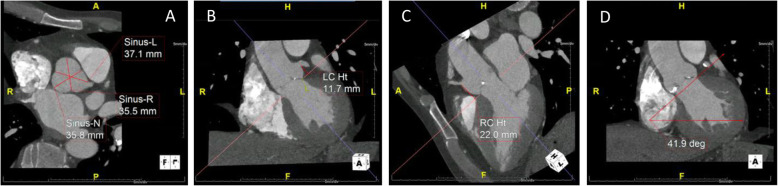
Multi-detector computer tomography (MDCT) example of **a** the sinuses of Valsalva; left sinus (Sinus-L), 37.1 mm, right sinus (Sinus-R), 35.5 mm and non-coronary sinus (Sinus-N), 35.8 mm; **b** the distance of the ostium of the left coronary artery from the aortic annulus (LC Ht), 11.7 mm; **c** the distance of the ostium of the right coronary artery from the aortic annulus (RC Ht), 22.0 mm; and **d** aortic root angle, 41.9°

**Fig. 6:**
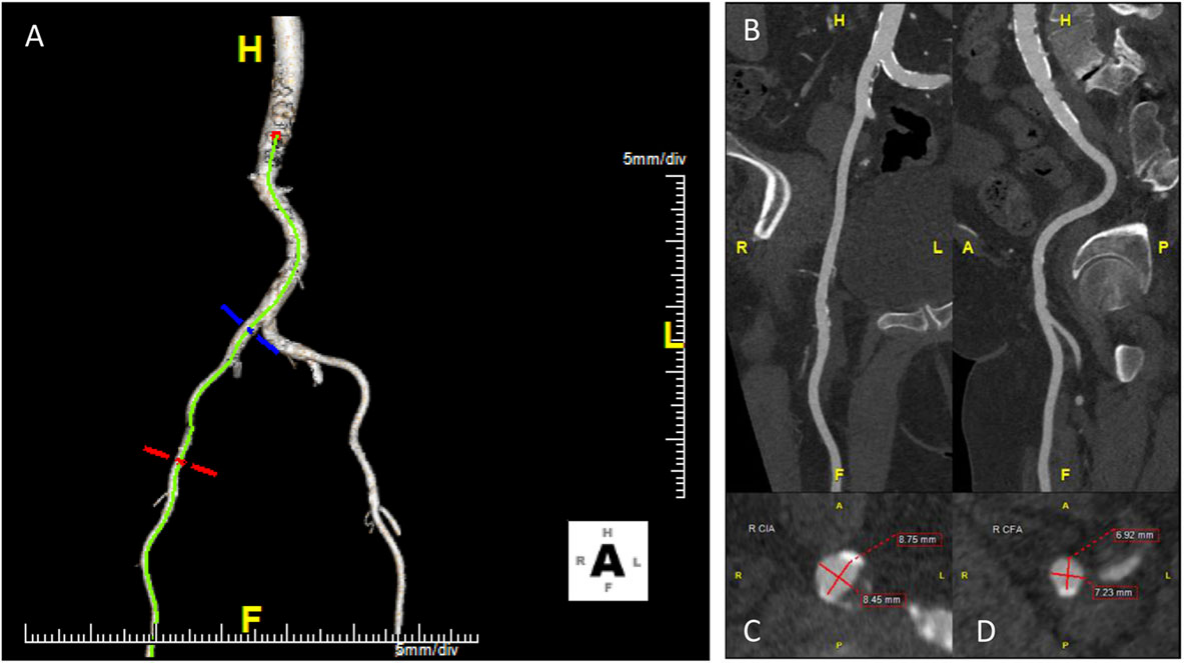
Multi-detector computer tomography (MDCT) example of **a** abdominal aorta; **b** right iliofemoral artery, long-axis; **c** right iliac artery, short-axis, 8.75 mm × 8.45 mm; and **d** right femoral artery, short-axis, 6.92 mm × 7.23 mm

MDCT (example provided in Figs. 4, 5, and 6 with corresponding hypothetical results in Tables 2 and 3) is playing an increasingly important role in the pre-procedural evaluation of TAVR patients and has become a core element of the standard imaging pathway for all TAVR patients. From delineating the anatomy and pathophysiology of the aortic valvular complex, to assessing the safest vascular access routes, MDCT has surpassed more traditional imaging modalities including echocardiography, angiography, and magnetic resonance. In this section, we will consider the use of MDCT during the pre-procedural assessment and planning for TAVR deployment by regions, namely the aortic valvular complex and aortic annulus, coronary arteries, the thoraco-abdominal aorta, and iliofemoral vasculature.

**Table 2:** Aortic annular and LVOT dimensions

	Aortic annulus	LVOT
Average diameter	27.2 mm	27.3 mm
Perimeter	86.9 mm	87.0 mm
Area	5.83 cm^2^	5.84 cm^2^

*LVOT* left ventricular outflow tract

**Table 3:** Dimensions of sinuses of Valsalva and coronary artery height from the aortic annulus

Coronary artery height	
Left	11.7 mm
Right	22.0 mm
Sinuses of Valsalva	
Left coronary sinus	37.1 mm
Right coronary sinus	35.5 mm
Non-coronary sinus	35.8 mm

Electrocardiogram (ECG)-gated acquisition of the aortic valvular complex, including the aortic annulus, will limit motion artifact and substantially improve overall image quality. Depending on the patient’s age and comorbidities, and reasons to limit the use of intravenous contrast, CT acquisition can be limited to the aortic root if necessary. Otherwise, most TAVR protocols call for an ECG-gated material-enhanced MDCT with a scanning range similar to that used for CT imaging of the coronary arteries (Salgado et al. 2014). As the dimensions of the aortic valvular complex, and in particular the aortic annulus, vary throughout the cardiac cycle, the annular cross-sectional diameter, both long and short axis, circumference, and area, should be measured when largest, during the systolic phase. Investigators have shown up to a 5-mm difference in annular dimensions between the systolic and diastolic phase of the cardiac cycle in young, healthy volunteers (de Heer et al. 2011), while others have demonstrated minimal variation in patients with severe aortic valve calcification (Tops et al. 2008; Shiran et al. 2009; Bertaso et al. 2012; Hamdan et al. 2012). Annular area and perimeter have become the predominant measurements used for valve sizing. Additionally, two-dimensional echocardiography does not consistently appreciate the ellipsoid shape of the aortic root, including the left ventricular outflow tract and aortic annulus. Any overestimation of the size of the aortic annulus places the patient at risk of aortic root injury or aortic annular disruption during implantation. There is a similar tendency to underestimate the sizing of the TAVR prosthesis when the diastolic dimensions of the aortic valvular complex are considered, increasing the risk of paravalvular aortic regurgitation after deployment (Jilaihawi et al. 2012; Willson et al. 2012; Blanke et al. 2012).

There is increasing evidence that the presence, extent and distribution of calcification along the aortic valvular complex, or “landing zone” of the TAVR prosthesis is associated with intra- and postprocedural complications including paravalvular aortic regurgitation (Marwan et al. 2013; Zegdi et al. 2008) and conduction abnormalities (Maeno et al. 2017), inadequate balloon expansion and subsequent migration of the TAVR prostheses (Kapadia et al. 2010), and displacement of the native aortic valve leaflets and subsequent occlusion of the coronary artery ostia (Thomas et al. 2010; Stabile et al. 2010). MDCT has emerged as the superior modality for assessing and quantifying aortic valvular complex calcification (Jilaihawi et al. 2014; Jilaihawi et al. 2016) and can be quantified in 2–3 mm slices using non-contrast CT. The Agatston score, originally developed to quantify coronary artery calcium scoring, is also used to quantify aortic valvular complex and device landing zone scoring (Agatston et al. 1990). When a non-contrast CT is not used, a similar scoring system has been devised (Jilaihawi et al. 2014).

Accurate positioning of the TAVR prosthesis both along the angle of the aortic root and perpendicular to the native aortic annulus is imperative. Malpositioning of the prosthesis too high can result in embolization into the ascending aorta and occlusion of the coronary ostia, while too low can result in embolization into the left ventricular cavity. The aorta-ventricular angle is important to consider because it can mean the TAVR prosthesis is not positioned perpendicularly in the aortic annulus. During deployment, it is crucial to maintain position such that “watermelon seeding” can occur, whereby the prosthesis dislodges distally into the aorta or proximally into the left ventricular cavity. In the event that the prosthesis does not dislodge proximally or distally, the risk of paravalvular regurgitation increases when the deployment angle is off-axis. Pre-procedural MDCT has supplanted repeated intra-procedural aortic root angiograms and has become the standard for defining the three-dimensional angle of the aortic root and determining optimal implantation angle or coplanar view prior to TAVR deployment (Leipsic et al. 2011; Gurvitch et al. 2010; Otsuka et al. 2007). More recently, three-dimensional angiographic reconstruction of the aortic root captured from rotational C-arm fluoroscopic images has been shown to be safe, practical, and accurate when compared with MDCT (Binder et al. 2012).

Understanding the anatomic relationship between the “landing zone” of any TAVR device and the structures of the aortic root is imperative for managing the associated risk of complications following deployment. MDCT has become the most valuable tool for assessing the anatomy and dimensions of the aortic root, including the sinuses of Valsalva, the height of the coronary ostia from the aortic annulus, and the dimensions of the left ventricular outflow tract, the sino-tubular junction in relation to the aortic annulus, and the ascending aorta. The entire aortic root anatomy defines how a TAVR device will be implanted. Defining the height of the aortic annulus to the coronary ostia can facilitate proper placement of the device, as well as predict the potential for complications. Coronary heights > 10 mm are generally considered safe when considering coronary obstruction. However, the sinuses of Valsalva are crucial for this determination. For instance, if the sinuses of Valsalva are large relative to the size of the TAVR prosthesis, there is often adequate space for the coronaries to fill during diastole, even if the coronary height is low. If there is a concern for coronary obstruction, this can influence valve choice. Self-expanding valves offer the ability to deploy the valve to ~ 80% and evaluate for coronary obstruction. If there are signs of obstruction, then the valve can be recaptured and repositioned or even removed. The self-expanding valve stent frame extends into the proximal ascending aorta, and therefore, the frame has to be crossed in order to re-access the coronaries. Balloon expandable valves sit lower in the aortic root and are less likely to interfere with coronaries during re-access, except in the case of low coronary heights. During valve deployment, the operators may choose to protect the coronaries by placing wires and even stents in place, such that they can be pulled back and deployed in the setting of obstruction (Ribeiro et al. 2013).

MDCT has also become useful for determining the atheromatous burden of the coronary vasculature, the patency of preexisting coronary artery bypass grafts, and left ventricle-to-chest wall position when a transapical approach is being considered. MDCT is limited for coronary artery assessment when calcium disease is advanced: in these cases, coronary angiography should be used to assess the presence and severity of coronary artery stenosis. CT imaging of the thoraco-abdominal aorta and iliofemoral vasculature has become the standard for planning safe and feasible vascular access prior to TAVR placement (Okuyama et al. 2015). Non-contrast MDCT can be used to assess overall peripheral vessel size, calcification, and tortuosity. Alternatively, non-gated magnetic resonance angiography (MRA) or intravascular ultrasound can be used in patients with reduced, but stable renal function. The concerted use of preprocedural CT imaging has led to a decrease in major and minor vascular complications and bleeding during TAVR procedures (Toggweiler et al. 2012; Achenbach et al. 2012).

## Cardiac MRI (CMR)

Non-contrast cardiac magnetic resonance (nc-CMR) imaging has become a safe and effective alternative imaging modality for assessing the aortic valvular complex in TAVR patients with underlying kidney dysfunction, and in whom intravenous contrast administration is contraindicated (Chen et al. 2015). Jabbour et al. demonstrated more accurate and reproducible assessment of aortic annular and root dimensions using CT or CMR when compared with two-dimensional transthoracic echocardiographic imaging (Jabbour et al. 2011). Shown to be superior for evaluating the ellipsoid nature of the aortic annulus and root, the use of CMR or CT prior to TAVR has been shown to result in a lower incidence of severe aortic regurgitation following device placement (Tops et al. 2008; Tzikas et al. 2011; Schultz et al. 2010). There is close agreement between CT and CMR for evaluating the aortic annulus and root dimensions, and therefore, CMR is a reliable alternative imaging modality in patients in whom contrast CT imaging is contraindicated or when CT is not available (Jabbour et al. 2011; Pontone et al. 2013). In a series of 35 prospectively collected patients with severe aortic valve stenosis undergoing annular sizing for TAVR device placement, Gopal et al. showed no significant propensity-adjusted difference in perimeter, area, and average, maximum, or minimum diameters between nc-CMR and CT when measured during diastole (Gopal et al. 2015). The authors report similar results when dimensions were measured at the end of systole, with one notable exception. The minimum annulus diameter was significantly smaller with nc-CMR. The authors conclude that in patients in whom contrast administration is contraindicated or not advisable, nc-CMR provides an accurate alternative to CT for aortic annular sizing.

CMR has also been shown to be valuable in assessing the severity of aortic stenosis, regurgitation, or mixed stenotic/regurgitant lesions (Caruthers et al. 2003). Accuracy of CMR imaging can be impacted by the imaging plane relative to the aortic annulus and flow jet (Chai and Mohiaddin 2005), as well as the amount of calcium on the aortic valvular complex. In addition to offering an alternative imaging modality in patients with renal disease, a more physiologic orifice area can be calculated for the aortic valve and left ventricular outflow tract flow using CMR compared with CT that can only measure an anatomic aortic valve orifice area (Pibarot and Dumesnil 2012b). The severity of low-flow, low-gradient aortic valve stenosis, as well as right and left ventricular volumes and ejection fraction can be accurately assessed using CMR before and during dobutamine stress test (O'Sullivan et al. 2015).

CMR imaging of peripheral vasculature, namely the ilio-femoral vessels, is severely limited when vascular calcification is present. While MRI angiography is used to determine vessel dimensions, this imaging technique requires the use of gadolinium contrast, predisposing patients with severe renal dysfunction to nephrogenic systemic fibrosis. Non-contrast CMR is still investigational (Renker et al. 2016), and alternative imaging modalities should be used to assess the peripheral vasculature.

Of note, currently used TAVR prostheses are MRI conditional, meaning patients who have undergone prior TAVR device placement are not necessarily excluded from entering an MRI machine for that reason (see the Institute for Magnetic Resonance Safety, Education and Research website for details regarding each device (MRIsafety.com n.d.)). Similarly, patients with a permanent pacemaker may have conditional devices that do not exclude them from MR imaging. MRI-conditional pacing leads and boxes, however, are only approved for scanning at 1.5-T field strength (Rogers and Waksman 2016).

## Conclusion

Minimally invasive aortic valve replacement therapy is quickly becoming a viable alternative to open surgical approaches. Safe and accurate pre-procedural assessment of cardiovascular anatomy, physiology, and pathophysiology prior to TAVR procedures can mean the difference between success and catastrophic failure. Moreover, caring for patients undergoing TAVR procedures, or any cardiac procedure for that matter, is best done using a team model that includes cardiology valve experts, cardiovascular imaging experts, interventional cardiologists, cardiothoracic surgeons, cardiothoracic anesthesiologists, and valve clinic care coordinators. It therefore becomes imperative that team members share a basic understanding of the preprocedural imaging technologies available for optimizing the care of TAVR patients. Herein, we have reviewed current technology for assessing the anatomy, physiology, and pathophysiology of the aortic valvular complex, ventricular function, and peripheral vasculature, including echocardiography, cardiac catheterization, cardiac computed tomography, and cardiac magnetic resonance. While large, high-volume academic centers are continually pushing the frontiers of imaging technology and capabilities (Miller et al. 2016; Biaggi et al. 2015), and the multimodal imaging techniques outlined in this review are sufficient for safely and effectively caring for the vast majority of patients undergoing TAVR procedures. Our hope is that this review, albeit niche, will increase the familiarity and understanding of currently used imaging technologies for all team members caring for TAVR patients.

## Data Availability

N/A.

## Abbreviations

AS: Aortic valve stenosis
NYHA: New York Heart Association
GFR: Glomerular filtration rate
BMI: Body mass index
STS: Society of Thoracic Surgeons
SAVR: Surgical aortic valve replacement
TAVR: Transcatheter aortic valve replacement
AVC: Aortic valvular complex
TTE: Transthoracic echocardiogram
AVA: Aortic valve area
ASE: American Society of Echocardiography
LVOT: Left ventricular outflow tract
BSA: Body surface area
DI: Dimensionless index
AI: Aortic insufficiency
MR: Mitral regurgitation
CAD: Coronary artery disease
LF-LG: Low flow, low gradient
TEE: Transesophageal echocardiography
CO: Cardiac output
nc-CMR: Non-contrast cardiac MRI
PAp: Pulmonary artery pressure
MDCT: Multi-detector computed tomography
PCI: Percutaneous coronary intervention
